# A duality between utility transforms and probability distortions

**DOI:** 10.1007/s11238-025-10073-9

**Published:** 2025-07-23

**Authors:** Christopher P. Chambers, Peng Liu, Ruodu Wang

**Affiliations:** 1 Department of Economics, Georgetown University, Washington, USA; 2 School of Mathematics, Statistics and Actuarial Science, University of Essex, Colchester, UK; 3 Department of Statistics and Actuarial Science, University of Waterloo, Waterloo, Canada

**Keywords:** Distributional transforms, Probability distortions, Utility transforms, Rank-dependent-utility transforms, Quantiles

## Abstract

In this paper, we establish a mathematical duality between utility transforms and probability distortions. These transforms play a central role in decision under risk by forming the foundation for the classic theories of expected utility, dual utility, and rank-dependent utility. Our main results establish that probability distortions are characterized by commutation with utility transforms, and utility transforms are characterized by commutation with probability distortions. These results require no additional conditions, and hence each class can be axiomatized with only one property. Moreover, under monotonicity, rank-dependent utility transforms can be characterized by set commutation with either utility transforms or probability distortions.

## Introduction

Distributional transforms are mappings from one set of probability distributions to another set of distributions. They are widely used in economics, finance, and risk analysis. A classical example of a distributional transformation is the Lorenz curve (Lorenz, 1905; Gastwirth, 1971). Formally, we can think of a mapping carrying a distribution of wealth, represented by a cumulative distribution function (cdf), to another cdf over percentages. The Lorenz curve evaluated at a percentage *p* specifies the proportion of wealth held by *p* poorest individuals. The Lorenz curve has all of the properties of a cdf, and hence can be viewed as a cdf itself, rendering the Lorenz map a distributional transform. The recent study (Liu et al., 2021) contains a general treatment of distributional transforms with many other examples. Two special classes of distributional transforms, utility transforms and probability distortions, play a central role in decision theory. Informally, the former reflects the induced distribution of utils that a given distribution of wealth induces, whereas the latter is a classical representation device used in much of behavioral economics.

A very simple case of a decision model based on a distributional transform is the “dual theory” of Yaari (1987). The individual is supposed to base decisions over a pair of distributions on the expected values of the transformed distributions, rather than the distributions themselves. In risk management, this mapping is known as a distortion risk measure. Important special cases include both the Value-at-Risk and the Expected Shortfall, the two regulatory risk measures in banking and insurance (see, e.g., Föllmer & Schied, 2016; McNeil et al., 2015). In the same context, the distorted distribution can also be used to model tail risk (Liu & Wang, 2021). Recently, the dual theory has been implemented to study some classic economic problems; see e.g., Gershkov et al. (2022, 2023). Liu et al. (2021) axiomatized probability distortions using three conditions, which we summarize in Sect. 3.

The rank-dependent utility (RDU) model (Quiggin, 1982, 1993; Schmeidler, 1989) is one of the most common alternatives to the classical expected utility theory. It effectively generalizes Yaari’s dual theory by allowing both attitudes toward risk in the form of a utility index, as well as a distributional transform in the form of what is termed a “probability distortion.” Probability distortions are distributional transforms that work via transforming the cumulative probability of receiving some outcome according to some prespecified nondecreasing function. In this sense, the RDU model can be viewed as a composition of a utility transform and a probability distortion. Another related approach is Tversky and Kahneman (1992)’s cumulative prospect theory, which can be viewed as a combination of RDU for gains and losses, respectively.

A somewhat more recent decision theory that might also be understood as being based on distributional transforms is the theory of Bracha and Brown (2012), whereby an individual facing probabilistic risk is supposed to maximize an expected utility of some probability less a cost of “choosing” that probability. The cost is typically given as the relative entropy of the chosen probability to the true probability. This chosen probability can be viewed as a distributional transform.^1^ As such, this model is, in a sense, an objective version of the multiplier preferences first axiomatized in economics by Strzalecki (2011), themselves a special case of the variational preferences of Maccheroni et al. (2006).

Instead of considering distributional transforms model-by-model, in this work, we isolate the distributional transform from the underlying representations of interest. Given its ubiquity, we view the distributional transform as worthy of independent study. Our main goal here is to obtain a complete picture of which types of distributional transforms “commute” with respect to other classes of distributional transforms. This commutativity is interesting in a few different senses, as we will see later from our results. First, it has a concrete interpretation as invariance under a certain form of (non-linear) rescaling. Second, it helps to identify or characterize important classes of decision models. Third, it allows for convenient operations in applications on these popular distributional transforms. Fourth, and perhaps being the most elegant point of this paper, it offers a new duality between the expected utility theory and the dual theory.

Our first result seeks to understand for which distributional transforms is it the case that an ordinal rescaling of the input distribution results in the same ordinal rescaling of the output distribution.

For example, imagine that an outside observer (an economist) writes down a behavioral model involving distributional transforms, but wants to remain flexible about the timing of their application. For example, suppose it is known that a nonlinear income tax is present, but to the economist, only the distribution of pre-tax income is observable. It may be that the individual facing the risk possesses certain idiosyncratic tax credits or dues, unobservable to the economist.^2^ Thus, the individual faces a “true” distribution of ex-post income, which is unknown to the economist. In this case, rather than postulating a distribution over distributions, in the interest of parsimony, it would seem reasonable that the economist apply a distributional transform that results in the “correct” transformed distribution of ex-post incomes independently of what the tax rates are.^3^

In this example, the function carrying pre-tax income to post-tax income defines a distributional transform: a distribution over pre-tax income naturally induces a distribution over post-tax income. But we can think more generally of nondecreasing transformations. The transform may represent “utils,” which again may be unobservable to the economist. Or, perhaps such a transformation represents the discretization of income into a categorical variable, specifying which tax bracket the individual is in, and so forth.

To this end, we ask the basic question as to which distributional transforms commute with to every distributional transform induced by a nondecreasing function. In so doing, we provide a minimalist characterization of probability distortions in Sect. 3: A distributional transform is a probability distortion if and only if it commutes with every such nondecreasing function. Intuitively, this commutation property means that a (possibly non-strict) ordinal change in the input distribution leads to the same ordinal change in the output distribution. This result simplifies and extends the characterization in Liu et al. (2021).^4^

A natural question then presents itself. Probability transforms commute with respect to all ordinal changes in the input distribution. This in itself is a very powerful “robustness” condition. It then seems natural to ask whether they also potentially commute with respect to a *larger* class of transforms. We would then obtain a broader robustness result for free. For example, one natural class might be the class of all distributional transforms induced as “pushforward” measures for potentially non-monotone but measurable functions. It turns out that the answer here is negative, in a strong sense. For any distributional transform which does not arise from a nondecreasing function, there is a probability distortion that does not commute with respect to it. Thus, the monotone transforms are the largest class of distortions for which we may hope to achieve a natural robustness result for probability distortions.

This result is established in Sect. 4, where we establish that a distributional transform can be identified as a “utility transform” if it commutes with respect to the class of probability distortions. This commutation property means that a distortion of the input distribution leads to the same distortion of the output distribution. In a formal sense, these results can be understood as providing a duality between the class of utility transforms and probability distortions.^5^

The combination of results in Sects. 3 and 4 yields the crucial observation that probability distortions and utility transforms are characterized via commutation with each other. This observation lends support to the informal idea that Yaari’s theory is the (unique) natural dual version of the expected utility theory.

Finally, let us here discuss a third class of transforms; these are what we call the RDU transforms. These transforms are the composition of a probability distortion and a utility transform. We discuss RDU transforms in Sect. 5. These transforms do not in general commute with respect to arbitrary utility transforms. However, there is a sense in which they do commute. In particular, suppose we have given an RDU transform, based on a strictly increasing and surjective utility function. Then it can be shown that for any ordinal rescaling of the input distribution results in a *possibly different* ordinal rescaling of the output distribution. Similarly, any ordinal rescaling of the output distribution comes from a *possibly different* ordinal rescaling of the input distribution.

This property can be viewed as a commutativity property if we extend the distributional transform to a set-valued mapping.^6^ As a set-valued mapping, the set of utility transforms commutes with respect to the distributional transform.

It turns out that this property is characteristic of RDU transforms, under an additional hypothesis of monotonicity with respect to first order stochastic dominance. This property is clearly weaker than commutation with each utility transform, which requires the same utility transform on the input and the output distributions. A similar result holds if we replace utility transforms by probability distortions.

The main contributions of the paper are concise characterizations of utility transforms and RDU transforms, which were never characterized in the literature, as well as probability distortions, on which we improve over existing results. We admit that how these results can be applied to economic contexts is not fully clear. Some preliminary implications of our results for decision theory are discussed in Sect. 6.

For the most concise presentation, we focus on compactly supported distributions in the main part of the paper. Proofs of the main results in Sects. 3–5 are postponed to "Appendix A–D". Results in Sects. 3–5 are extended to other sets of utility and distortion functions in "Appendix E" and to general spaces of distributions in "Appendix F".

## The model

Let $\mathcal {M}$ be the set of compactly supported distributions on $\mathbb {R}$. A distribution in $\mathcal {M}$ will be identified with its cumulative distribution function (cdf). For a cdf *F*, we define its left quantile as

$$\begin{aligned} F_L^{-1}(t) = \inf \{x\in \mathbb {R}: F(x)\geqslant t\},~~t\in {(0,1]}, \end{aligned}$$

and its right quantile as

$$\begin{aligned} F_R^{-1}(t) = \inf \{x\in \mathbb {R}: F(x)> t\},~~t\in {[0,1)}. \end{aligned}$$

By increasing and decreasing, we mean in the non-strict sense.

A *distortion function (DF)* is an increasing function $d:[0,1]\rightarrow [0,1]$ with $d(0)=0$ and $d(1)=1$. A DF is also called a weighting function (e.g., Tversky & Kahneman, 1992). The set of all DFs is denoted by ${\mathcal {F}}_D$. A *utility function (UF)* is an increasing and continuous function $u:\mathbb {R}\rightarrow \mathbb {R}$. The set of all UFs is denoted by ${\mathcal {F}}_U$. For any increasing function *f*, write $f(x+)=\lim _{y\downarrow x}f(x)$ and $f(x-)=\lim _{y\uparrow x}f(x)$.

### Definition 1

(i) For $d\in {\mathcal {F}}_D$, the *probability distortion* generated by *d*, denoted by $T_d:\mathcal {M}\rightarrow \mathcal {M}$, is defined as $T_d(F)(x) =(d\circ F)(x+),~x\in \mathbb {R}$.
(ii) For $u\in {\mathcal {F}}_U$, the *utility transform* $T^u: {\mathcal {M}}\rightarrow {\mathcal {M}}$ is defined as a mapping from the distribution of *X* to the distribution of *u*(*X*), i.e., $T^u(F)=F\circ u^{-1},$ where *F* is treated as a measure on $\mathbb {R}$, and $u^{-1}(A)=\{x\in \mathbb {R}: u(x)\in A\}$ for any Borel measurable set $A\subseteq \mathbb {R}$.
(iii) For $T,T':\mathcal {M}\rightarrow \mathcal {M}$, we say that *T* *commutes* with $T'$ if $T\circ T'=T'\circ T$, where $\circ$ denotes composition.

Denote by $\mathcal {U}$ the set of continuous utility transforms: $\mathcal {U}=\{T^{u}:u\in {\mathcal {F}}_U\}$. Denote by ${\mathcal {D}}$ the set of probability distortions: ${\mathcal {D}}=\{T_d: d\in {\mathcal {F}}_D\}$.

### Remark 1

Some caution is required regarding the right limit in Definition 1 (i). A simple point is that taking a right limit renders $T_d(F)$ right-continuous, a requirement for a cdf. This is different from using a right-continuous version of *d*. For $d\in {\mathcal {F}}_D$, define $\widehat{d}$ as the right-continuous version of *d*, i.e., $\widehat{d}(x)=d(x+)$ for $x\in [0,1]$. Clearly $\widehat{d}$ may not be in ${\mathcal {F}}_D$, if for example $d(0+)>0$. Even if $\widehat{d}\in {\mathcal {F}}_D$, generally, $T_{\widehat{d}}\ne T_d$, as shown in the following simple example.

### Example 1

If *d* is not right-continuous, then there exists $x_0\in (0,1)$ such that $\widehat{d}(x_0)=d(x_0+)>d(x_0)$. Take $F = \textrm{Bernoulli}(1-x_0)$. Then $F(x)=x_0$ for $x\in [0,1)$. For $x\in [0,1)$, $T_d(F)(x)=(d \circ F)(x+)=d(x_0)<\widehat{d}(x_0)=T_{\widehat{d}}(F)(x)$, showing $T_{\widehat{d}}\ne T_d$.

The above discussion illustrates that, although a right limit is used in Definition 1 (i), it is not without loss to consider only right-continuous distortion functions. This subtle difference will be significant in the analysis of general distortion and utility functions, treated in "Appendix E".

## Probability distortions

Our first result characterizes probability distortions as the class of distributional transforms that commute with respect to every utility transform.

### Theorem 1

For a mapping $T: \mathcal {M}\rightarrow \mathcal {M}$, *T* commutes with each element of ${\mathcal {U}}$ if and only if $T\in {\mathcal {D}}$.

To interpret the above result, the commutation property requires that only the “ordinal” content of the input distribution matters for calculating the distributional transform *T*, in the sense that any ordinal rescaling of the input distribution results in the same ordinal rescaling of the output distribution under the distributional transform. The theorem then states that the distributional transform must necessarily be a probability distribution.

Theorem 1 is closely related to a characterization result in the literature, Theorem 1 of Liu et al. (2021), which states that for $T:\mathcal {M}\rightarrow \mathcal {M}$, *T* is monotone, lower-semicontinuous and commuting with each $T^u$ for all $u\in {\mathcal {F}}_U^\diamond$ if and only if $T=T_d$ for some right-continuous $d\in {\mathcal {F}}_D$, where ${\mathcal {F}}_U^\diamond$ is the set of all strictly increasing and continuous functions *u* satisfying $u(\mathbb {R})=\mathbb {R}$. To explain the terminology involved, for $F, G\in \mathcal {M}$, we write $F\leqslant _{\textrm{st}} G$ if $F(x)\geqslant G(x)$ for all $x\in \mathbb {R}$; *T* is *monotone* if $T(F)\leqslant _{\textrm{st}} T(G)$ for $F\leqslant _{\textrm{st}} G,~ F, G\in {\mathcal {M}}$; *T* is *lower-semicontinuous* if $T(F)\leqslant _{\textrm{st}} G\in {\mathcal {M}}$ whenever $\{F_n\}_{n\in \mathbb {N}}\subseteq {\mathcal {M}}$ converges weakly to $F\in {\mathcal {M}}$ and satisfies $T(F_n)\leqslant _{\textrm{st}} G$. In contrast to these properties, our results in Theorem 1 only require one condition to pin down probability distortions. Moreover, Theorem 1 characterizes more general probability distortions than that of Liu et al. (2021), as we do not have lower-semicontinuity. Below we present an example of a probability distortion *T* that is not lower-semicontinuous. This example shows that Theorem 1 is not only conceptually simpler than the corresponding result of Liu et al. (2021), but also covers more cases.

### Example 2

Define $T(F)=\delta _{F_R^{-1}(1/2)}$, where $\delta _x$ is the point mass distribution with probability 1 at *x*. Then, *T* is monotone and $T\circ T^u=T^u\circ T$ for all $u\in {\mathcal {F}}_D$. However, *T* does not satisfy lower semicontinuity. This can be checked as follows. Let $F_n= \textrm{Bernoulli}(1/2-1/n),~n\geqslant 2$, $F_0= \textrm{Bernoulli}(1/2)$ and $G= \mathrm U[0,1]$. Clearly, $F_n$ weakly converges to $F_0$ as *n* tends to infinity. By direct calculation, it follows that for $p\in (0,1)$,

$$\begin{aligned} q_p^T(F_n)=(F_{n})_R^{-1}(1/2)=0<p=G_L^{-1}(p), \end{aligned}$$

where $q_p^T(F)$ is the *p*-th left quantile of *T*(*F*), implying $T(F_n)\leqslant _{\textrm{st}} G$. However,

$$\begin{aligned} q_p^T(F_0)=(F_{0})_R^{-1}(1/2)=1>p=G_L^{-1}(p),~p\in (0,1), \end{aligned}$$

implying that $T(F_0)\leqslant _{\textrm{st}} G$ does not hold. Hence *T* is not lower-semicontinuous. Applying Theorem 1, *T* is still a probability distortion in the sense of (i) of Definition 1. Let $d(x)=\mathbbm {1}_{(1/2,1]}(x),~x\in [0,1]$. Note that $d\in {\mathcal {F}}_D$ but *d* is not right-continuous. Moreover,

$$\begin{aligned} T_d(F)(x)=d\circ F(x+)=\mathbbm {1}_{[ F_R^{-1}(1/2),\infty )}(x)=T(F)(x),~x\in \mathbb {R}, \end{aligned}$$

and this verifies that $T=T_d$.

The fact that probability distortions can be characterized with only one property may be surprising, and it is built on a related result in the recent literature. Our proof of Theorem 1 uses Fadina et al. (2023, Theorem 2), where they showed that a mapping from ${\mathcal {M}}$ to $\mathbb {R}$ commutes with utility transforms if and only if it is a quantile functional.

## Utility transforms

In a parallel fashion to Sect. 3, we characterize utility transforms via commutation with probability distortions. This result establishes that utility transforms are the “maximal” class of distributional transforms with respect to which each probability distortion commutes.

### Theorem 2

For a mapping $T: \mathcal {M}\rightarrow \mathcal {M}$, *T* commutes with each element in $\mathcal {D}$ if and only if $T\in \mathcal {U}$.

Theorem 2 establishes that for a distributional transform *T*, if distorting the input distribution is the same as distorting the output distribution with the same probability distortion, then the distributional transform is a probability distortion.

Although being nicely parallel with Theorem 1, the idea of pinning down utility transforms via probability distortions in Theorem 2 is novel. We are not aware of any results in the literature that characterize utility transforms. Mathematically, the proof of Theorem 2 does not follow directly from that of Theorem 1, because the roles of probability distortions (changing the distribution function) and utility transforms (changing the quantile functon) are not exactly symmetric.

Theorems 1 and 2 offer a mathematical duality between the expected utility theory and the dual utility theory of Yaari (1987), in the sense that one can be derived from the other. Taking the class of increasing and continuous utility functions as given, if we look at the expected value of any transform that commutes with these utility transforms, then we arrive at a dual utility. Conversely, taking the the class of all distortion functions as given, if we look at the expected value of any transform that commutes with these probability distortions, then we arrive at an expected utility. This connection is new to the literature.

The separation of marginal utility (modelled by UF) and the probabilistic risk (modelled by DF) attitudes under RDU (see Definition 2 in Sect. 5) are considered by Wakker (1994), where these attitudes are characterized independently of each other to allow for comparison of risk attitudes across decision makers. This perspective is different from Theorems 1 and 2, where utility transforms and probability distortions are mutually determined by commutation with each other.

### Remark 2

We discuss an application of our characterization results. When dealing with model misspecification, two forms of distributional transforms are commonly considered. First, agents are often assumed to simplify a complex objective distribution over outcomes into the best fitting distribution within a restricted set of possible data generating processes. Often, this is done by mapping the true distribution into the one that minimize the relative entropy, or another measure of divergence, within a simpler mental model of the agents; see e.g., Esponda and Pouzo (2016). Second, as agents do not trust the model completely, they can use a variational multiplier evaluation as in Hansen and Sargent (2001), that is, effectively, an exponential transformation of the utility. Since minimization of relative entropy is in general not a probability distortion Theorems 1 and 2 imply that the order of these operations cannot be exchanged. Trying to use the reversed order might be tempting, but its decision-theoretic foundation is unclear.

## RDU transforms

In the two sections above, we have characterized both the class of utility transforms and the class of probability distortions. A natural question is whether an RDU transform, which is a composition of a utility transform and a probability distortion, also admits a characterization in a similar fashion. We address this question in this section.

We first formally define an RDU transform.

### Definition 2

A distributional transform $T:\mathcal {M}\rightarrow \mathcal {M}$ is an *RDU transform* if there exist $d\in {\mathcal {F}}_D$ and $u\in {\mathcal {F}}_U$ such that $T=T_d\circ T^u$. Here, *u* is called the UF of *T* and *d* is called the DF of *T*.

To understand special properties of RDU transforms, we first observe that an RDU transform does not commute with utility transforms in general, as implied by Theorem 1. Essentially, this is because utility transforms do not commute with each other; for instance, $2(x+1)$ is not equal to $2x+1$. The same non-commutation of RDU transforms holds also with probability distortions. Therefore, in order to characterize RDU transforms, we need to seek for weaker properties than commutation with utility transforms (or probability distortions). Although a utility transform $T^{u_1}$ with $u_1\in {\mathcal {F}}_U$ does not commute with another one $T^{u_2}$, assuming that $u_1$ is strictly monotone and surjective (i.e., $u_1(\mathbb {R})=\mathbb {R}$), there exists $u_3\in {\mathcal {F}}_U$ such that

1 $$\begin{aligned} T^{u_3}\circ T^{u_1}= T^{u_1} \circ T^{u_2}; \text{ equivalently, } u_3\circ u_1= u_1 \circ u_2, \end{aligned}$$

and such $u_3$ is given by $u_3 = u_1 \circ u_2 \circ u_1^{-1}$. This inspires to use the property of set commutation. For a set ${\mathcal {T}}$ of functions and a function *T*, we write ${\mathcal {T}}\circ T = \{T'\circ T: T'\in {\mathcal {T}}\}$, and similarly, $T\circ {\mathcal {T}} = \{T\circ T': T'\in {\mathcal {T}}\}$.

### Definition 3

A distributional transform $T:\mathcal {M}\rightarrow \mathcal {M}$ is said to *commute* with a set ${\mathcal {T}}$ of distributional transforms on ${\mathcal {M}}$ if ${\mathcal {T}}\circ T=T\circ {\mathcal {T}}$.

Stated equivalently, ${\mathcal {T}}\circ T=T\circ {\mathcal {T}}$ means that for any $T_R\in {\mathcal {T}}$ there exists $T_L\in {\mathcal {T}}$ such that $T_L\circ T=T\circ T_R$, and for any $T_L\in {\mathcal {T}}$ there exists $T_R\in {\mathcal {T}}$ such that $T_L\circ T=T\circ T_R$. Clearly, commutation with the set ${\mathcal {T}}$ is weaker than commutation with each element of ${\mathcal {T}}$, which further requires $T_L=T_R$ in both statements.

### Theorem 3

Let $T:\mathcal {M}\rightarrow \mathcal {M}$ be a monotone distributional transform. Then *T* commutes with $\mathcal {U}$ if and only if $T=T_d\circ T^u$, where *u* is strictly increasing and surjective.

The reason why the UF of *T* in Theorem 3 needs to be strictly increasing and surjective can be seen from the above discussion; we can check that both properties of $u_1$ are needed for the existence of $u_3$ satisfying (1) for each $u_2$ and the existence of $u_2$ satisfying (1) for each $u_3$.

Set commutation with ${\mathcal {T}}$ has a similar interpretation to commutation in Sect. 4 as the effect of rescaling the input and the output distributions, but now the output rescaling can be different from the input one; yet these two scaling operations belong to the same class $\mathcal {U}$. If we remove monotonicity of *T* in Theorem 3, we will include mappings like $T=T_d\circ T^u$ with strictly decreasing and surjective *u* in the conclusion of Theorem 3, which is undesirable in decision theory.

The duality between utility transforms and probability distortions hints at a similar result to Theorem 3 formulated with probability distortions. This intuition checks out but it requires some technical work to formalize. For this, we need to consider a set smaller than ${\mathcal {D}}$. Let ${\mathcal {F}}_U^L$ be the set of all increasing and left-continuous functions on $\mathbb {R}$ and let ${\mathcal {F}}_D^R$ be the set of all right-continuous functions in ${\mathcal {F}}_D$. Note that ${\mathcal {F}}_U\subseteq {\mathcal {F}}_U^L$ and ${\mathcal {F}}_D^R\subseteq {\mathcal {F}}_D$. The utility transform can be naturally extended to ${\mathcal {F}}_U^L$ and the RDU can also be extended to the case with increasing and left-continuous UF. We denote $\mathcal {U}^L=\{T^{u}:u \in {\mathcal {F}}_U^L\}$ and ${\mathcal {D}}^R=\{T_d: d\in {\mathcal {F}}_D^R\}$.

### Theorem 4

Let $T:\mathcal {M}\rightarrow \mathcal {M}$ be a monotone distributional transform. Then *T* commutes with $\mathcal {D}^R$ if and only if $T=T_d\circ T^u$, where *u* is left-continuous and *d* is strictly increasing and continuous.

The interpretation of Theorem 4 is similar to that of Theorem 3. Roughly, RDU transforms are precisely those which are “exchangeable" (in the sense of $T\circ {\mathcal {T}}={\mathcal {T}}\circ T$) with either scaling or distortion as a whole class.

## Discussions

Our results offer a fundamental symmetry between utility transforms and probability distortions. In spirit, this is similar to a duality between the expected utility (EU) theory of von Neumann and Morgenstern (1947) and the dual utility (DU) theory of Yaari (1987). Nevertheless, the duality between EU and DU is based on parts of the representation, rather than behavior. EU assumes the independence axiom (among others) to arrive at the preference representation $F\mapsto \int u(x)\textrm{d}F(x)$ for some utility function $u \in {\mathcal {F}}_U$. DU assumes a dual-independence axiom (plus the same other axioms) to arrive at the preference representation $F\mapsto \int x \textrm{d}(d\circ F)(x)$ for some distortion function $d\in {\mathcal {F}}_D$. Although conceptually plausible, it is not clear how one could start from the independence axiom to derive the dual independence axiom, and vice versa. Our results in Theorems 1 and 2 gives a way to obtain from utility transforms to probability distortions, and also the other way around. Since EU and DU are represented by the expected value of the transformed risk distribution, our results also suggest that EU and DU can be mathematically derived from each other.

Regarding RDU transforms, there are also some interesting observations. Let ${\mathcal {R}}$ be the class of RDU transforms of the form $T_d\circ T^{u}$ where $d\in {\mathcal {F}}_D$ and $u\in {\mathcal {F}}_U$. First, note that the order of the composition of $T_d$ and $T^{u}$ for an RDU transform does not matter, justified by either Theorems 1 or 2. Second, due to the same reason, the class ${\mathcal {R}}$ is closed under composition, and so are $\mathcal {U}$ and ${\mathcal {D}}$. Therefore, ${\mathcal {R}}$ equipped with composition is a semigroup with ${\mathcal {U}}$ and ${\mathcal {D}}$ being its sub-semigroups. Moreover, ${\mathcal {R}}$ is the smallest semigroup containing both ${\mathcal {U}}$ and ${\mathcal {D}}$. This, from an algebraic perspective, may justify that RDU is the most natural generalization of EU and DU compared to all other ways, such as a linear combination or a maximum of EU and DU (which are obviously strange).

Next, we rephrase our results in the context of decision making. Assume that a decision maker has a unique *perception*, which describes how she perceives a distribution in ${\mathcal {M}}$ presented to her, and her preference is represented by the mean of her perception. Certainly, the perception is precisely a distributional transform on ${\mathcal {M}}$.

We assume that the decision maker is described by her perception, instead of a preference relation as in the classic literature. We say that a decision maker is an EU thinker if her perception is a utility transform in $\mathcal {U}$ (thus, the preference is EU); she is a DU thinker if her perception is a probability distortion in ${\mathcal {D}}$ (thus, the preference is DU); she is an RDU thinker if her perception is the composition of a utility transform in $\mathcal {U}$ with a strictly increasing and surjective UT and a probability distortion in ${\mathcal {D}}$ (thus, the preference is RDU in Theorem 3). The next proposition is immediate from Theorems 1–3.

### Proposition 1

For a decision maker equipped with a monotone perception,

(i) she is a DU thinker if and only if her perception commutes with each EU perception;
(ii) she is an EU thinker if and only if her perception commutes with each DU perception;
(iii) she is an RDU thinker if and only if her perception commutes with the set of EU perceptions.

A decision maker with perception *T* perceives a distribution *F* as $G= T(F)$. Commuting with EU perceptions mathematically means that she perceives $F\circ u^{-1}$ as $G\circ u^{-1}$ for all $u\in {\mathcal {F}}_U$. This means intuitively that her perception is independent of the metric or scale (e.g., linear, log, exponential, etc) used for the underlying random outcome. On the other hand, commuting with DU perceptions mathematically means that she perceives $d \circ F$ as $d\circ G$ for all $d\in {\mathcal {F}}_D$. In other words, her perception is independent of a probabilistic weighting. These explanations are actually quite obvious: a DU thinker applies a distortion *d* to a (cumulative) probability *p* regardless of its outcome value, and an EU thinker applies a utility *u* to an outcome *x* regardless of its probability. Putting Proposition 1 into the above context, the independence principle above alone is able to characterize both DU and EU thinkers, and a slight variation is able to characterize RDU thinkers.

As for future research, let $\mathcal {T}_0$ be the set of all distributional transforms, i.e., mappings from $\mathcal {M}$ to $\mathcal {M}$. We may define $\mathcal {C}:2^{{\mathcal {T}}_0} \rightarrow 2^{{\mathcal {T}}_0}$ by $\mathcal {C}(\mathcal {T})=\bigcap _{T'\in \mathcal {T}}\{T\in {\mathcal {T}}_0:T\circ T'=T'\circ T\}$, that is, the set of elements of $\mathcal {T}_0$ that commute with respect to each element of $\mathcal {T}$. Hence, $\mathcal {C}$ forms an (antitone) Galois connection with itself with respect to set inclusion on $2^{\mathcal {T}_0}$ (see e.g., Blyth 2005).^7^ This implies that $\mathcal {C}^2:=\mathcal {C}\circ \mathcal {C}$ forms a closure operator, and the results in Theorems 1 and 2 indicate that the sets $\mathcal {U}$ and $\mathcal {D}$ are closed sets according to this operator, with the further property that $\mathcal {U}=\mathcal {C}({{\mathcal {D}}})=\mathcal {C}^2({{\mathcal {U}}})$ and $\mathcal {D}=\mathcal {C}(\mathcal {U})=\mathcal {C}^2({{\mathcal {D}}})$. This latter property is a formal expression of the term “duality”. A general investigation of the map $\mathcal {C}$, together with its implied lattice of closed sets, could be fruitful.

## Data Availability

No data are used in the study.

## Proof of Theorem 1

For $p\in (0,1]$, let $q_p:\mathcal {M}\rightarrow \mathbb {R}$ be the left quantile at probability level *p*, that is, $q_p(F)=F^{-1}_L(p)$. Further, for $T: \mathcal {M}\rightarrow \mathcal {M}$, define $q^T_p: \mathcal {M}\rightarrow \mathbb {R}$ by $q^T_p(F)= q_p(T(F))$. For an increasing function *u*, let $u_R^{-1}(x)=\sup \{y\in \mathbb {R}: u(y)\leqslant x\}$ with $\sup \emptyset =-\infty$. For notational convenience, we extend the domain of $F\in \mathcal {M}$ to $[-\infty ,\infty ]$ by letting $F(-\infty )=0$ and $F(\infty )=1$.

### Proof of Theorem 1

We first prove the “if” part; that is, we will show that $T_d$ and $T^u$ commute. For $d\in {\mathcal {F}}_D$, let $T(F)(x)=T_d(F)(x)=(d\circ F)(x+),~x\in \mathbb {R}$. Note that for any $F\in \mathcal {M}$ and $u\in {\mathcal {F}}_U$,

$$\begin{aligned} T_d\circ T^u(F)(x)=[d\circ (F\circ u_R^{-1})](x+)=\lim _{y\downarrow x}d(F(u_R^{-1}(y))), \end{aligned}$$

and

$$\begin{aligned} T^u\circ T_d (F)(x)=w(u_R^{-1}(x)), \end{aligned}$$

where $w(x)=(d \circ F)(x+)=\lim _{y\downarrow x}d(F(y))$ for $x\in {\mathbb {R}}$ and $w(+\infty )=1, w(-\infty )=0.$ Moreover, as $u\in {\mathcal {F}}_U$, if $u_R^{-1}(x)\in \mathbb {R}$, then $u_R^{-1}(y)>u_R^{-1}(x)$ for $y>x$. Hence, $\lim _{y\downarrow x}d(F(u_R^{-1}(y)))=w(u_R^{-1}(x))$, implying $T_d\circ T^u(F)(x)=T^u\circ T_d (F)(x).$ For $u_R^{-1}(x)=\infty$, by definition, we have $T_d\circ T^u(F)(x)=T^u\circ T_d (F)(x)=1.$ For $u_R^{-1}(x)=-\infty$, we have $\lim _{y\downarrow x}u_R^{-1}(y)=-\infty$, which implies $T_d\circ T^u(F)(x)=T^u\circ T_d (F)(x)=d(0)=0.$ Consequently, $T_d\circ T^u=T^u\circ T_d$.

Next, we show the “only if” part. For each $p\in (0,1)$, we claim that the functional $q^T_p: \mathcal {M}\rightarrow \mathbb {R}$ satisfies $q^T_p(T^u(F)) = u(q^T_p(F))$ for all $u\in {\mathcal {F}}_U$.

Using the commutation of *T* and $T^u$ for $u\in {\mathcal {F}}_U$ and the fact that $q_p$ commutes with $T^u$ for $u\in {\mathcal {F}}_U$, we have

$$\begin{aligned} q^T_p(T^u(F)) = q_p (T \circ T^u (F)) = q_p ( T^u\circ T (F)) = u(q_p( T (F))) = u(q^T_p(F)). \end{aligned}$$

By Theorem 2 of Fadina et al. (2023), $q^T_p$ is a (left or right) quantile of *F* at a fixed level for all $F\in \mathcal {M}$. We denote this level by $g(p)\in [0,1]$. Hence there exists $E\subseteq (0,1)$ such that for any $F\in \mathcal {M}$

2 $$\begin{aligned} q_p^T(F)=\left\{ \begin{array}{cc} F_L^{-1}(g(p)),& ~p\in E\\ F_R^{-1}(g(p)),& ~p\in (0,1)\setminus E \end{array}\right. . \end{aligned}$$

Note that by definition, $p\notin E$ if $g(p)=0$ and $p\in E$ if $g(p)=1$ . Clearly, *g*(*p*) is an increasing function over (0, 1). By (2), we have

3 $$\begin{aligned} T(F)(x)&=\lambda \left( \{p\in E: F_L^{-1}(g(p))\leqslant x\}\cup \{p\in (0,1)\setminus E: F_R^{-1}(g(p))\leqslant x\}\right) \nonumber \\&=\lambda \left( \{p\in E: g(p)\leqslant F(x)\}\cup \{p\in (0,1)\setminus E: F_R^{-1}(g(p))\leqslant x\}\right) , ~x\in \mathbb {R}, \end{aligned}$$

where $\lambda$ is the Lebesgue measure on [0, 1]. Note that for $F(x)\in (0,1)$

$$\begin{aligned} \{p\in (0,1)\setminus E: F_R^{-1}(g(p))\leqslant x\}=\left\{ \begin{array}{cc} \{p\in (0,1)\setminus E: g(p)< F(x)\},& x<F_R^{-1}(F(x))\\ \{p\in (0,1)\setminus E: g(p)\leqslant F(x)\},& x=F_R^{-1}(F(x)). \end{array}\right. \end{aligned}$$

Hence

4 $$\begin{aligned} d(F(x)-)\leqslant T(F)(x)\leqslant d(F(x)),~x\in (F_R^{-1}(0), F_L^{-1}(1)), \end{aligned}$$

where $d(x)=\lambda (\{p\in (0,1): g(p)\leqslant x\})$ is a right-continuous and increasing function with $d(1)=1$. Note that $d(0)\in [0,1]$. Moreover, by (3) we have

5 $$\begin{aligned} T(F)(x)=\left\{ \begin{array}{cc} 0,& x<F_R^{-1}(0)\\ 1,& x\geqslant F_L^{-1}(1), \end{array}\right. \end{aligned}$$

and for $x=F_R^{-1}(0)$, $T(F)(x)=d(0)$ if $F(F_R^{-1}(0))=0$; $d(F(x)-)\leqslant T(F)(x)\leqslant d(F(x))$ if $F(F_R^{-1}(0))>0$. Let *D* be the set of all discontinuous points of *d* on (0, 1). For $x\in D$, let

$$\begin{aligned} r(x):=\lambda \left( \{p\in E: g(p)\leqslant x\}\cup \{p\in (0,1)\setminus E: g(p)<x\}\right) . \end{aligned}$$

Noting that $d(x-)\leqslant r(x)\leqslant d(x),~x\in D$, it follows that the function ${\widehat{d}}$ given by

$$\begin{aligned} \widehat{d}(x)=\left\{ \begin{array}{cc} d(x),& x\in (0,1]\setminus D\\ r(x),& x\in D\\ 0,& x=0 \end{array} \right. \end{aligned}$$

is an increasing function on [0, 1] with $\widehat{d}(0)=0$ and $\widehat{d}(1)=1$. Note that $\widehat{d}$ may not be right-continuous but $\widehat{d}\in {\mathcal {F}}_D$. We next show that $\widehat{d}$ is the desirable distortion function such that $T=T_{\widehat{d}}$. It follows from (4) that

$$\begin{aligned} T(F)(x)=\widehat{d}(F(x)),~F(x)\in (0,1)\setminus D. \end{aligned}$$

For $$F(x)=c\in D$$, if $$x<F_R^{-1}(F(x))$$,

$$\begin{aligned} T(F)(x)=\lambda \left( \{p\in E: g(p)\leqslant c\}\cup \{p\in (0,1)\setminus E: g(p)<c\}\right) =r(c)=\widehat{d}(F(x)). \end{aligned}$$

For $F(x)=c\in D$ and $x=F_R^{-1}(F(x))$, we have $T(F)(x)=d(c)$, which may not be equal to $\widehat{d}(F(x))$. Hence, $T(F)(x)\ne \widehat{d}(F(x))$ holds over $(F_R^{-1}(0),F_L^{-1}(1))$ only if $F(x)\in D$ and $x=F_R^{-1}(F(x))$. This implies that $T(F)(x)=\widehat{d}(F(x))$ does not hold over $(F_R^{-1}(0),F_L^{-1}(1))$ only at countable number of points. Note that $(\widehat{d}\circ F)(x+)$ is the right-continuous version of $\widehat{d}(F(x))$ over $\mathbb {R}$. Hence, by monotonicity and the right continuity of the two functions, we have

$$\begin{aligned} T(F)(x)=(\widehat{d}\circ F)(x+),~x\in [F_R^{-1}(0),F_L^{-1}(1)). \end{aligned}$$

Moreover, by (5), we have

$$\begin{aligned} T(F)(x)=\widehat{d}(F(x))=(\widehat{d}\circ F)(x+),~x\in (-\infty , F_R^{-1}(0))\cup [F_L^{-1}(1),\infty ). \end{aligned}$$

Combing the above results, we conclude $T(F)(x)=(\widehat{d}\circ F)(x+)$ for all $x\in \mathbb {R}.$ This completes the proof of Theorem 1. $\square$

## Proof of Theorem 2

### Proof of Theorem 2

The “if” part is the same as Theorem 1. We focus on the “only if" part. For $n\geqslant 1$, denote $\mathcal {C}_n=\{F\in \mathcal {M}: F(-n)=0,~F(n)=1\},$ and let $F_{U_n}$ represent the uniform distribution on $[-n,n]$. Clearly,

$$\begin{aligned} \mathcal {C}_n=\{T_d(F_{U_n}): d~\text {is right-continuous and }~d\in {\mathcal {F}}_D\},~\mathcal {C}_n\subseteq \mathcal {C}_{n+1}, n\geqslant 1, ~\text {and}~\mathcal {M}=\bigcup _{n=1}^\infty \mathcal {C}_n. \end{aligned}$$

By $T\circ T_d=T_d\circ T$, it follows that for $p\in (0,1)$ and $F=T_d(F_{U_n})$ with a right-continuous $d\in {\mathcal {F}}_D$

$$\begin{aligned} q_p^T(F)=q_p^T(T_d(F_{U_n}))=q_p^{T_d}(T(F_{U_n}))=q_{d_L^{-1}(p)}^T(F_{U_n}). \end{aligned}$$

Note that $d_L^{-1}(p)=( {F_L^{-1}(p)+n})/({2n}),~p\in (0,1)$ and $d_L^{-1}(p)>0$ for $p\in (0,1)$. Define $u_n:(-n,n]\rightarrow \mathbb {R}$ by $u_n(t)=q_{(t+n)/2n}^T(F_{U_n})$. Then, for $F\in \mathcal {C}_n$,

$$\begin{aligned} q_p^T(F)=u_n(F_L^{-1}(p)),~p\in (0,1). \end{aligned}$$

Setting $F=F_{U_n}$, it follows that for $n\geqslant 1$ and $m\geqslant 0$,

$$\begin{aligned} q_p^T(F_{U_n})=u_n(-n+2np)=u_{n+m}(-n+2np),~p\in (0,1). \end{aligned}$$

Hence, for $n\geqslant 1$ and $m\geqslant 0$, we have $u_{n+m}(t)=u_n(t),~t\in (-n,n)$. Moreover, one can verify $u_{n+m}(n)=u_n(n)$. Therefore, we can define a function $u:\mathbb {R}\rightarrow \mathbb {R}$ by letting $u(t)=u_n(t),~t\in (-n,n]$. Clearly, *u* is an increasing and left-continuous function. Moreover, we have for $F\in \mathcal {M}$

6 $$\begin{aligned} q_p^T(F)=u(F_L^{-1}(p)),~p\in (0,1). \end{aligned}$$

This implies that for any $F\in \mathcal {M}$, $T(F)=T^{u}(F)$.

We next show continuity of *u*. We assume by contradiction that there exists $x_0\in \mathbb {R}$ such that $u(x_0+)>u(x_0)$. Denote $y_0=u(x_0)$ and without loss of generality, we assume $x_0\in (0,1)$. Let $d(x)=\mathbbm {1}_{(x_0,1]}(x),~x\in [0,1]$ and $F_U$ be the uniform distribution on [0, 1]. Note that $d\in {\mathcal {F}}_D$ and $(d \circ F_U)(x+)=\mathbbm {1}_{[x_0,\infty )}(x)$. Hence

$$\begin{aligned} T^{u}\circ T_d(F_U)(y_0)=\mathbbm {1}_{[x_0,\infty )}(u_R^{-1}(y_0))=\mathbbm {1}_{[x_0,\infty )}(x_0)=1, \end{aligned}$$

and

$$\begin{aligned} T_d\circ T^{u}(F_U)(y_0)=\lim _{y\downarrow y_0}d(F_U(u_R^{-1}(y)))=d(F_U(x_0))=d(x_0)=0. \end{aligned}$$

This implies that $T^{u}\circ T_d\ne T^{u}\circ T_d$ for some $d\in {\mathcal {F}}_D$, yielding a contradiction. Hence *u* is continuous. This completes the proof. $\square$

## Proof of Theorem 3

In order to prove Theorem 3, we need an additional result for mappings $\rho :\mathcal {M}\rightarrow \mathbb {R}$. We say that $\rho :\mathcal {M}\rightarrow \mathbb {R}$ commutes with the set ${\mathcal {U}}$ if ${\mathcal {F}}_U\circ \rho =\rho \circ {\mathcal {U}}$, where ${\mathcal {F}}_U\circ \rho =\{u\circ \rho : u\in {\mathcal {F}}_U\}$ and $\rho \circ {\mathcal {U}}=\{\rho \circ T: T\in {\mathcal {U}}\}$. For a mapping $\rho :\mathcal {M}\rightarrow \mathbb {R}$, we say $\rho$ is a *left quantile* if there exists some $p\in (0,1]$ such that $\rho (F)=F_L^{-1}(p)$ for all $F\in \mathcal {M}$; $\rho$ is a *right quantile* if there exists some $p\in [0,1)$ such that $\rho (F)=F_R^{-1}(p)$ for all $F\in \mathcal {M}$; $\rho$ is a *quantile* if $\rho$ is either a left or right quantile.

### Proposition 2

For a mapping $\rho :\mathcal {M}\rightarrow \mathbb {R}$, $\rho$ commutes with ${\mathcal {U}}$ if and only if there exists a strictly monotone, continuous and surjective *h* such that $\rho =h\circ \widehat{\rho }$, where $\widehat{\rho }:\mathcal {M}\rightarrow \mathbb {R}$ is a quantile.

### Proof

We first consider the “if” part. For $u\in {\mathcal {F}}_U$, let $\psi =h\circ u\circ h^{-1}$. Note that $\psi \in {\mathcal {F}}_U$. Using the commutation of utility transforms and quantiles (Theorem 2 of Fadina et al. (2023)), we have

$$\begin{aligned} \psi \circ \rho =\psi \circ h\circ \widehat{\rho }=h\circ u\circ \widehat{\rho }=h\circ \widehat{\rho }\circ T^u=\rho \circ T^u. \end{aligned}$$

Let $\omega =h^{-1}\circ u\circ h$. Clearly, $\omega \in {\mathcal {F}}_U$. Analogously as above, we have

$$\begin{aligned} \rho \circ T^{\omega }=h\circ \widehat{\rho }\circ T^{\omega }=h\circ \omega \circ \widehat{\rho }=u\circ h\circ \widehat{\rho }=u\circ \rho . \end{aligned}$$

Note that in the above equations, we only use the commutation of $\widehat{\rho }$ and increasing transforms. Hence $\rho$ commutes with ${\mathcal {U}}$.

We next show the “only if” part. By ${\mathcal {U}}$-commutation, we have for any $u\in {\mathcal {F}}_U$, there exists $\psi , \omega \in {\mathcal {F}}_U$ such that $\psi \circ \rho (\delta _x)=\rho \circ T^u(\delta _x)$ and $\rho \circ T^{\omega }(\delta _x)=u\circ \rho (\delta _x)$. We denote $h(x)=\rho (\delta _x),~x\in \mathbb {R}$. Then direct computation yields $\psi \circ h=h\circ u$ and $h\circ \omega =u\circ h$. This implies *h* is continuous and strictly monotone and $h(\mathbb {R})=\mathbb {R}$. The proof is given below.

We assume by contradiction that $h(\mathbb {R})$ has a lower or upper bound, i.e., $h(\mathbb {R})\subseteq [n_1,\infty ]$ for some $n_1\in \mathbb {R}$, or $h(\mathbb {R})\subseteq (-\infty ,n_2]$ for some $n_2\in \mathbb {R}$. Without loss of generality, we consider the case $h(\mathbb {R})\subseteq (-\infty ,n_2]$ for $n_2\in \mathbb {R}$. Let $u\in {\mathcal {F}}_U$ be such that $u(h(0))>n_2+1$. Note that for any $\omega \in {\mathcal {F}}_U$, $h\circ \omega (0)\leqslant n_2<u\circ h(0)$. This means that $h\circ \omega =u\circ h$ does not hold for all $\omega \in {\mathcal {F}}_U$, leading to a contradiction. Hence $h(\mathbb {R})$ is neither bounded from below nor from above. If *h* is not monotone, then there exist $a, b, c\in \mathbb {R}$ with $a<b<c$ such that $h(a)<h(b)$ and $h(b)>h(c)$, or $h(a)>h(b)$ and $h(b)<h(c)$. For the former case, let $u\in {\mathcal {F}}_U$ be such that $u(a)=b$ and $u(b)=c$. It follows that $h\circ u(a)>h\circ u(b)$. Moreover, by the fact that there exists $\psi \in {\mathcal {F}}_U$ such that $\psi \circ h=h\circ u$, we have $\psi \circ h(a)>\psi \circ h(b)$, which contradicts with $h(a)<h(b)$ and $\psi \in {\mathcal {F}}_U$. The latter case is not possible by the same reasoning. Hence, *h* is a monotone function. Next, we assume *h* is flat over [*a*, *b*] with $a,b\in \mathbb {R}$ and $a<b$. As $h(\mathbb {R})$ is not a singleton, there exist $c,d\in \mathbb {R}$ with $c<d$ such that $h(c)\ne h(d)$. Let $u\in {\mathcal {F}}_U$ be such that $u(a)=c$ and $u(b)=d$. Hence we have $h\circ u(a)\ne h\circ u(b)$. In contrast, $\psi \circ h(a)=\psi \circ h(b)$ for all $\psi \in {\mathcal {F}}_U$, leading to a contradiction. Thus, *h* is strictly monotone. Next, assume by contradiction that *h* is not continuous on $\mathbb {R}$. Then there exist $x_0, x_1\in \mathbb {R}$ such that *h* is not continuous at $x_0$ but continuous at $x_1$. Let $u:x\mapsto x-x_1+x_0,~x\in \mathbb {R}$. It follows that $u\in {\mathcal {F}}_U$ and $h\circ u$ is not continuous at $x_1$. However, $\psi \circ h$ is continuous at $x_1$ for all $\psi \in {\mathcal {F}}_U$, which contradicts with the fact that there exists $\psi \in {\mathcal {F}}_U$ such that $\psi \circ h=h\circ u$. Hence *h* is continuous on $\mathbb {R}$. Combining all the properties proved above, we conclude that *h* is continuous, strictly monotone and satisfying $h(\mathbb {R})=\mathbb {R}$.

Using the above conclusion, $\psi \circ h=h\circ u$ implies $\psi =h\circ u\circ h^{-1}$. Hence,

$$\begin{aligned} \psi \circ \rho =h\circ u\circ h^{-1}\circ \rho =\rho \circ T^u, \end{aligned}$$

which further implies $u\circ h^{-1}\circ \rho =h^{-1}\circ \rho \circ T^u$. Let $\widehat{\rho }=h^{-1}\circ \rho$. It follows that $\widehat{\rho }:\mathcal {M}\rightarrow \mathbb {R}$ and $u\circ \widehat{\rho }=\widehat{\rho }\circ T^u$ for all $u\in {\mathcal {F}}_U$. Using Theorem 2 of Fadina et al. (2023), we obtain that $\widehat{\rho }$ is a quantile. We further have $\rho =h\circ \widehat{\rho }$. $\square$

The result in Proposition 2 leads to the proof of Theorem 3.

### Proof of Theorem 3

The “if” part follows by checking the definition. Note that $T=T_d\circ T^{h}$ for $d\in {\mathcal {F}}_D$ and $h\in {\mathcal {F}}_U^\diamond$. For $u\in {\mathcal {F}}_U$, let $\psi =h\circ u\circ h^{-1}\in {\mathcal {F}}_U$. It follows from Theorem 1 that

$$\begin{aligned} T^{\psi }\circ T=T^{\psi }\circ T_d\circ T^{h}=T_d\circ T^{\psi }\circ T^{h}=T_d\circ T^{\psi \circ h}=T_d\circ T^{h\circ u}=T\circ T^u. \end{aligned}$$

Moreover, let $\omega =h^{-1}\circ u\circ h\in {\mathcal {F}}_U$. Analogously, we have

$$\begin{aligned} T\circ T^{\omega }=T_d\circ T^{h}\circ T^{\omega }=T_d\circ T^{h\circ \omega }=T_d\circ T^{u\circ h}=T^u\circ (T_d\circ T^{h})= T^u\circ T. \end{aligned}$$

Hence *T* commutes with ${\mathcal {U}}$.

We next focus on the “only if" part. Recall that for $p\in (0,1)$, $q^T_p: \mathcal {M}\rightarrow \mathbb {R}$ is given by $q^T_p(F)=(T(F))_L^{-1}(p)$. Note that for any $u\in {\mathcal {F}}_U$, there exist $\psi , \omega \in {\mathcal {F}}_U$ such that $T^{\psi }\circ T=T\circ T^u$ and $T\circ T^{\omega }=T^u\circ T$. This implies that $\psi \circ q^T_p=q^T_p\circ T^u$ and $q^T_p\circ T^{\omega }=u\circ q^T_p$ for all $p\in (0,1)$. Hence, $q^T_p$ commutes with ${\mathcal {U}}$ for all $p\in (0,1)$. By Proposition 2 and monotonicity of *T*, for $p\in (0,1)$, there exists $h_p\in {\mathcal {F}}_U^\diamond$ such that $q^T_p=h_p\circ \widehat{\rho }_p$, where $\widehat{\rho }_p$ is a quantile. Note that $\psi \circ q^T_p(\delta _x)=q^T_p\circ T^u(\delta _x)$ for all $x\in \mathbb {R}$ and $p\in (0,1)$. Hence we have $\psi \circ h_p=h_p\circ u$ for all $p\in (0,1)$. A simple manipulation yields that $h_{p_1}\circ u\circ h_{p_1}^{-1}=h_{p_2}\circ u\circ h_{p_2}^{-1}$ holds for all $u\in {\mathcal {F}}_U$ and $p_1, p_2\in (0,1)$, which is equivalent to $h_{p_2}^{-1}\circ h_{p_1}\circ u=u\circ h_{p_2}^{-1}\circ h_{p_1}$ for all $u\in {\mathcal {F}}_U$ and $p_1, p_2\in (0,1)$. Denote $f=h_{p_2}^{-1}\circ h_{p_1}$. Then $f\in {\mathcal {F}}_U^\diamond$ and $f\circ u=u\circ f$ for all $u\in {\mathcal {F}}_U$. Let $u(x)=\min (x, b),~x\in \mathbb {R}$ for some $b\in \mathbb {R}$. It follows that for $x\geqslant b$, $f\circ u(x)=f(b)$ and $u\circ f(x)=\min (f(x), b)$. Hence $f(b)=\min (f(x), b)$ for $x\geqslant b$. Letting $x\rightarrow \infty$, we have $f(b)=b$ for $b\in \mathbb {R}$, which means that *f* is the identity function. Hence $h_{p_1}=h_{p_2}$ for all $p_1, p_2\in (0,1)$. Let $h=h_{1/2}$. Hence, we have for all $p\in (0,1)$, $q_p^T=h\circ \widehat{\rho }_p$ with $h\in {\mathcal {F}}_U^\diamond$.

Note that $q_p^T(\delta _x)=h\circ \widehat{\rho }_p(\delta _x)=h(x)$ for all $p\in (0,1)$. Therefore, $T(\delta _x)=\delta _{h(x)}$ for $x\in \mathbb {R}$. Using the fact that *T* commutes with ${\mathcal {U}}$, we have $T^{\psi }\circ T(\delta _x)=T\circ T^u(\delta _x)$, implying $\psi \circ h=h\circ u$, and yielding $\psi =h\circ u \circ h^{-1}$. It follows from $T^{\psi }\circ T=T\circ T^u$ that $T^{h}\circ T^u\circ T^{h^{-1}}\circ T=T\circ T^u$, implying $T^u\circ (T^{h^{-1}}\circ T)=(T^{h^{-1}}\circ T)\circ T^u$. Letting $\widehat{T}=T^{h^{-1}}\circ T$, we have $\widehat{T}$ commutes with $T^u$ for all $u\in {\mathcal {F}}_U$. In light of Theorem 1, we conclude that there exists $d\in {\mathcal {F}}_D$ such that $\widehat{T}=T_d$. Hence $T=T^{h}\circ T_d=T_d\circ T^{h}$.$\square$

## Proof of Theorem 4

### Proof of Theorem 4

We first show the “if part”. Suppose there exist $u\in {\mathcal {F}}_U^L$ and $d\in {\mathcal {F}}_D^R$ such that $T=T_d\circ T^u$, where *d* is strictly increasing and continuous. Note that for $d\in {\mathcal {F}}_D^R$, $T_d(F)(x)=d(F(x)),~x\in \mathbb {R}$. Hence, for $d\in {\mathcal {F}}_D^R$ and $u\in {\mathcal {F}}_U^L$

$$\begin{aligned} T_d\circ T^u(F)(x)=d(F(u_R^{-1}(x)))=T^u\circ T_d(F)(x),~x\in \mathbb {R}, \end{aligned}$$

indicating that $T^u$ and $T_d$ commute. For any $T_{d_1}\in {\mathcal {D}}^R$, let $d_2=d\circ d_1\circ d^{-1}$, where $d^{-1}$ is the inverse function of *d*. Clearly, $d_2\in {\mathcal {F}}_D^R$. Therefore, we have

$$\begin{aligned} T_{d_2}\circ T=T_{d_2}\circ T_d\circ T^u=T_{d_2\circ d}\circ T^u=T_{d\circ d_1}\circ T^u=T_{d}\circ T_{d_1}\circ T^u=T_{d}\circ T^u \circ T_{d_1}=T\circ T_{d_1}. \end{aligned}$$

Moreover, by letting $d_2=d^{-1}\circ d_1\circ d\in {\mathcal {F}}_D^R$, we have

$$\begin{aligned} T\circ T_{d_2}=T_d\circ T^u\circ T_{d_2}=T_d\circ T_{d_2}\circ T^u=T_{d\circ d_2}\circ T^u=T_{d_1\circ d}\circ T^u=T_{d_1}\circ T_d\circ T^u=T_{d_1}\circ T. \end{aligned}$$

We next show the “only if” part. Let $B_{y,z}^{\alpha }=\alpha \delta _y+(1-\alpha )\delta _z$ with $y<z$ and $\alpha \in [0,1]$. We consider two different scenarios.

First, we suppose there exist $y_0<z_0$, $x_0\in \mathbb {R}$ and $\alpha _0\in (0,1)$ such that $T(B_{y_0,z_0}^{\alpha _0})(x_0)\in (0,1)$. Let $g(\alpha )=T(B_{y_0,z_0}^{\alpha })(x_0),~\alpha \in [0,1]$. Note that monotonicity of *T* implies that *g* is an increasing function. Moreover, commutation with ${\mathcal {D}}^R$ means that for any $d_1\in {\mathcal {F}}_D^R$, there exist $d_2, d_3\in {\mathcal {F}}_D^R$ such that $T_{d_2}\circ T=T\circ T_{d_1}$ and $T\circ T_{d_3}=T_{d_1}\circ T$. By the second equality, we have $T\circ T_{d_3}(B_{y_0,z_0}^{\alpha })(x_0)=T_{d_1}\circ T(B_{y_0,z_0}^{\alpha })(x_0)$, which can be rewritten as $g\circ d_3=d_1\circ g$. Using the fact that $g(\alpha _0)\in (0,1)$, we have $[0,1]=\{d_1(g(\alpha _0)): d_1\in {\mathcal {F}}_D^R\}\subseteq \{g(\alpha ):\alpha \in [0,1]\}$. It follows from the fact $g(\alpha )\in [0,1]$ for $\alpha \in [0,1]$ that $\{g(\alpha ):\alpha \in [0,1]\}=[0,1]$. Hence $g\in {\mathcal {F}}_D^R$ is continuous.

By the first equality, we have $T_{d_2}\circ T(B_{y_0,z_0}^{\alpha })(x_0)=T\circ T_{d_1}(B_{y_0,z_0}^{\alpha })(x_0)$, which can be simplified as $d_2\circ g=g\circ d_1$. We next show that *g* is strictly increasing. Suppose by contradiction that there exist $0< \alpha _1<\alpha _2<\alpha _3<1$ such that $g(\alpha _1)=g(\alpha _2)<g(\alpha _3)$ or $g(\alpha _1)<g(\alpha _2)=g(\alpha _3)$. For the former case, let $d_1\in {\mathcal {F}}_D$ satisfy $d_1(\alpha _1)=\alpha _2$ and $d_1(\alpha _2)=\alpha _3$. This implies $g\circ d_1(\alpha _1)\ne g\circ d_1(\alpha _2)$. For any $d_2\in {\mathcal {F}}_D^R$, we have $d_2\circ g(\alpha _1)=d_2\circ g(\alpha _2)$, which is contradicted by the existence of $d_2\in {\mathcal {F}}_D^R$ such that $d_2\circ g=g\circ d_1$. For the latter case, we can show a similar contradiction. Hence *g* is strictly increasing. Therefore, we conclude that $g\in {\mathcal {F}}_D^R$ is strictly increasing and continuous.

By $d_2\circ g=g\circ d_1$, we have $d_2=g\circ d_1\circ g^{-1}$, where $g^{-1}$ is the inverse function of *g*. It follows from $T_{d_2}\circ T=T\circ T_{d_1}$ that $T_{g\circ d_1\circ g^{-1}}\circ T=T_g\circ T_{d_1}\circ T_{g^{-1}}\circ T=T\circ T_{d_1}$. We denote $\widehat{T}=T_{g^{-1}}\circ T$. It follows that $T_{d_1}\circ \widehat{T}=\widehat{T}\circ T_{d_1}$ for all $T_{d_1}\in {\mathcal {D}}^R$. This implies (6) in the proof of Theorem 2. Hence, we have $\widehat{T}=T^u$ for some $u\in {\mathcal {F}}_U^L$, which further implies $T=T_g\circ T^u$ for some $u\in {\mathcal {F}}_U^L$ and some strictly increasing and continuous $g\in {\mathcal {F}}_D^R$.

Next, we consider the case that $T(B_{y,z}^{\alpha })(x)\in \{0,1\}$ for all $y<z$, $x\in \mathbb {R}$ and $\alpha \in (0,1)$. Using $T\circ T_{d_3}(\delta _y)=T_{d_1}\circ T(\delta _y)$, we have $T(\delta _y)=T_{d_1}\circ T(\delta _y)$ for any $d_1\in {\mathcal {F}}_D^R$. This implies $T(\delta _y)=\delta _{u(y)}$ for some function *u*. Hence for $\alpha =0, 1$, $T(B_{y,z}^{\alpha })(x)\in \{0,1\}$ also holds. We next show that for fixed *x*, *y*, *z*, $T(B_{y,z}^{\alpha })(x)=0$ for all $\alpha \in [0,1]$ or $T(B_{y,z}^{\alpha })(x)=1$ for all $\alpha \in [0,1]$. Suppose by contradiction that there exist $0<\alpha _1<\alpha _2\leqslant 1$ such that $T(B_{y,z}^{\alpha _1})(x)=0$ and $T(B_{y,z}^{\alpha _2})(x)=1$. Let $d_1\in {\mathcal {F}}_D^R$ such that $d_1(\alpha _1)=\alpha _2$. Then we have $T\circ T_{d_1}(B_{y,z}^{\alpha _1})(x)=T(B_{y,z}^{\alpha _2})(x)=1$. However, for any $d_2\in {\mathcal {F}}_D^R$, $T_{d_2}\circ T(B_{y,z}^{\alpha _1})(x)=0$, which contradicts the existence of $d_2\in {\mathcal {F}}_D^R$ such that $T_{d_2}\circ T=T\circ T_{d_1}$. For $0\leqslant \alpha _1<\alpha _2<1$ such that $T(B_{y,z}^{\alpha _1})(x)=0$ and $T(B_{y,z}^{\alpha _2})(x)=1$, we can similarly show a contradiction. Hence, $T(B_{y,z}^{\alpha })(x)=0$ for all $\alpha \in [0,1]$ or $T(B_{y,z}^{\alpha })(x)=1$ for all $\alpha \in [0,1]$. This implies $T(B_{y,z}^{0})=T(B_{y,z}^{1})$ for all $y<z$, i.e., $T(\delta _y)=T(\delta _z)$ for all $y<z$. Using the fact $T(\delta _y)=\delta _{u(y)}$, we have $T(\delta _y)=\delta _{c}$ for all $y\in \mathbb {R}$ and some $c\in \mathbb {R}$, which together with monotonicity of *T* implies $T=T^c$. Hence, we have $T=T_d\circ T^c$, where $d\in {\mathcal {F}}_D^R$ is strictly increasing and continuous. This completes the proof. $\square$

## General sets of utility and distortion functions

In this appendix, we study probability distortions and utility transforms on different sets of UF and DF from the ones in Theorems 1, 2 and 3. We use the notation introduced in Sect. 5. The main intuition is that, for the commutation property, requiring more continuity in the distortion functions results in less continuity in the utility functions, and the same holds true if the positions of distortion functions and utility functions are switched. Therefore, the next result, which is a different version of Theorems 1 and 2, further illustrates the symmetry between utility transforms and probability distortions.

### Proposition 3

For a mapping $$T: \mathcal {M}\rightarrow \mathcal {M}$$,

(i) *T* commutes with each element of $\mathcal {U}^L$ if and only if $T\in {\mathcal {D}}^R$;
(ii) *T* commutes with each element of $\mathcal {D}^R$ if and only if $T\in \mathcal {U}^L$.

### Proof

First note that the “if” parts of (i)-(ii) are implied by the commutation of $T_d$ and $T^u$, which has been shown in the proof of Theorem 4.

We next focus on the “only if’ part of (i). Note that the commutation of *T* and each element in $\mathcal {U}^L$ implies the commutation of *T* and each element in $\mathcal {U}$. By Theorem 1, we have $T=T_d$ for some $d\in {\mathcal {F}}_D$. Next we show $d\in {\mathcal {F}}_D^R$ by contradiction. Suppose that there exists $x_0\in [0,1)$ such that $d(x_0)<d(x_0+)$. Moreover, let $F_U$ be the standard uniform distribution on [0, 1], and

$$\begin{aligned} u(x)=\left\{ \begin{array}{cc} x,& x\leqslant x_0\\ x+1,& x>x_0\\ \end{array} \right. . \end{aligned}$$

Then we have $u\in {\mathcal {F}}_U^L$. Direct computation gives

$$\begin{aligned} T_d\circ T^u(F_U)(x_0)=[d\circ (F_U\circ u_R^{-1})](x_0+)=\lim _{y\downarrow x_0}d(F_U(u_R^{-1}(y)))=d(x_0), \end{aligned}$$

and

$$\begin{aligned} T^u\circ T_d (F_U)(x_0)=w(u_R^{-1}(x_0))=w(x_0)=d(x_0+), \end{aligned}$$

where $w(x)=(d \circ F_U)(x+)=\lim _{y\downarrow x}d(F_U(y))$. This implies $T_d\circ T^u\ne T^u\circ T_d$, leading to a contradiction. Hence, $d\in {\mathcal {F}}_D^R$, and we establish the “only if" part of (i).

The “only if” part of (ii) is indicated by (6) in the proof of Theorem 2. $\square$

## General spaces of distributions

Let $\mathcal {M}_0$ be the set of all distributions on ${\mathbb {R}}$. In this appendix, we consider the distributional transforms $T:\mathcal {M}_0\rightarrow \mathcal {M}_0$. Both probability distortions and utility transforms are naturally extended from $\mathcal {M}$ to $\mathcal {M}_0$. We obtain the results of Theorems 1-2 and Proposition 3 for distributional transforms defined on $\mathcal {M}_0$. Let $\widehat{{\mathcal {F}}_D}$ be the set of all increasing functions $d:[0,1]\rightarrow [0,1]$ with $d(0)=d(0+)=0$ and $d(1)=d(1-)=1$. We denote $\widehat{{\mathcal {D}}}=\{T_d: d\in \widehat{{\mathcal {F}}_D}\}$. In the next proposition, we derive some characterizations of probability distortions and utility transforms defined on $\mathcal {M}_0$.

### Proposition 4

For a mapping $$T: \mathcal {M}_0\rightarrow \mathcal {M}_0$$,

(i) *T* commutes with each element of ${\mathcal {U}}$ if and only if $T\in \widehat{{\mathcal {D}}}$;
(ii) *T* commutes with each element of $\widehat{{\mathcal {D}}}$ if and only if $T\in {\mathcal {U}}$.

### Proof

The “if” parts of both (i) and (ii) follow from the same arguments as in the proof of Theorem 1. We next focus on the “only if" parts.

(i) First note that $T\circ T^u=T^u\circ T$ for all bounded $u\in {\mathcal {F}}_U$ implies that $T(F)\in \mathcal {M}$ for all $F\in \mathcal {M}$. Hence, in light of Theorem 1, there exists $d\in {\mathcal {F}}_D$ such that for all $F\in \mathcal {M}$,

$$\begin{aligned} T(F)(x)=(d \circ F)(x+),~x\in \mathbb {R}. \end{aligned}$$

We next show that this equality holds for all $F\in \mathcal {M}_0$. Let $u(x)=\arctan (x),~x\in \mathbb {R}$. Then for any $F\in \mathcal {M}_0$, we have $T^u(F)\in \mathcal {M}$. Hence for $F\in \mathcal {M}_0$

$$\begin{aligned} T\circ T^u(F)(x)=[d \circ (F\circ u_R^{-1})](x+)=\lim _{y\downarrow x}d(F(u_R^{-1}(y)))=w(u_R^{-1}(x)), \end{aligned}$$

and $T^u\circ T(F)(x)=(T(F))(u_R^{-1}(x)),$ where $w(x)=\lim _{y\downarrow x}d(F(y))$. Using the fact that $T\circ T^u=T^u\circ T$ and $u_R^{-1}((-\pi /2, \pi /2))=\mathbb {R},$ we have $T(F)(x)=w(x)=T_d(F)(x),~x\in \mathbb {R}.$ Hence $T(F)=T_d(F)$ for all $F\in \mathcal {M}_0$. Taking $F(x)= e^x \wedge 1,~x\in \mathbb {R}$, it follows that

$$\begin{aligned} d(0+)=\lim _{x\rightarrow -\infty }d(e^x\wedge 1)=\lim _{x\rightarrow -\infty }T(F)(x)=0. \end{aligned}$$

Moreover, letting $F(x)= (1-e^{-x})\mathbbm {1}_{\{x\geqslant 0\}},~x\in \mathbb {R}$, we analogously obtain $d(1-)=1$. Consequently, $d\in \widehat{{\mathcal {F}}_D}$. We establish claim (i).

(ii) We denote $F_0(x)=\frac{1}{2}+\frac{1}{\pi }\arctan (x),~x\in \mathbb {R}$ and note that $F_0\in \mathcal {M}_0$. For $F\in \mathcal {M}_0$, letting $d(x)=F((F_0)_R^{-1}(x)),~x\in (0,1)$ with $d(0)=0$ and $d(1)=1$, it follows that *d* is right-continuous, $d\in \widehat{{\mathcal {F}}_D}$ and $T_d(F_0)=F$. Hence,

$$\begin{aligned} \mathcal {M}_0=\{T_d(F_0): d~\text {is right-continuous and}~d\in \widehat{{\mathcal {F}}_D}\}. \end{aligned}$$

For $F=T_d(F_0)$ with right-continuous $d\in \widehat{{\mathcal {F}}_D}$, using $T\circ T_d=T_d\circ T$, we have for $p\in (0,1)$

$$\begin{aligned} q_p^T(F)=q_p^T(T_d(F_0))=q_p^{T_d}(T(F_0))=q_{d_L^{-1}(p)}^T(F_0). \end{aligned}$$

Note that for $p\in (0,1)$, $d_L^{-1}(p)=F_0(F_L^{-1}(p))$ and $d_L^{-1}(p)\in (0,1)$. Letting $u(x)=q_{F_0(x)}^T(F_0)$, we have

$$\begin{aligned} q_p^T(F)=u(F_L^{-1}(p)),~p\in (0,1),~\text {and}~u\in {\mathcal {F}}_U^L, \end{aligned}$$

implying $T=T^{u}$. We can show the continuity of *u* using the same argument as in the proof of Theroem 2. $\square$

We next obtain a version of Proposition 3 for distributional transforms defined on $\mathcal {M}_0$.

### Proposition 5

For a mapping $$T: \mathcal {M}_0\rightarrow \mathcal {M}_0$$,

(i) *T* commutes with each element of $\mathcal {U}^L$ if and only if $T\in {\mathcal {D}}^R\cap \widehat{{\mathcal {D}}}$;
(ii) *T* commutes with each element of $\mathcal {D}^R\cap \widehat{{\mathcal {D}}}$ if and only if $T\in \mathcal {U}^L$.

### Proof

The “if” parts of both (i) and (ii) follow from the same arguments as in the proof of Proposition 3. We next focus on the “only if" parts.

(i) By Proposition 4, there exists $d\in \widehat{{\mathcal {F}}_D}$ such that for all $F\in \mathcal {M}_0$,

$$\begin{aligned} T(F)(x)=(d \circ F)(x+),~x\in \mathbb {R}. \end{aligned}$$

Using the same argument as in the proof of (i) of Proposition 3, we can show that *d* is right-continuous. Hence $T=T_d\in {\mathcal {D}}^R\cap \widehat{{\mathcal {D}}}$. The “only if” part of (ii) follows from the same argument as in the proof of (ii) of Proposition 4. Hence the detail is omitted. $\square$

We define the RDU transform on $\mathcal {M}_0$ as follows: a distributional transform $T:\mathcal {M}_0\rightarrow \mathcal {M}_0$ is an RDU transform if there exist $d\in \widehat{{\mathcal {F}}_D}$ and $u\in {\mathcal {F}}_U^L$ such that $T=T_d\circ T^u$.

With this definition of RDU, the results in Theorems 3-4 remain true for the mappings $T:\mathcal {M}_0\rightarrow \mathcal {M}_0$.

### Proposition 6

Let $T:\mathcal {M}_0\rightarrow \mathcal {M}_0$ be a monotone distributional transform. The following hold:

(i) *T* commutes with ${\mathcal {U}}$ if and only if *T* is an RDU transform with strictly increasing and surjective UF;
(ii) *T* commutes with $\mathcal {D}^R\cap \widehat{{\mathcal {D}}}$ if and only if *T* is an RDU transform with left-continuous UF and strictly increasing and continuous DF.

### Proof

(i) First note that Proposition 2 also holds for $\rho :\mathcal {M}_0\rightarrow \mathbb {R}$. All the rest of the proof follows the same argument as in the proof of Theorem 3. We omit the details.

(ii) The “if” part follows from checking the definition, which is exactly the same as the proof of Theorem 4. We next show the “only if" part, which only requires some refinement of the proof of Theorem 4. Along with the proof of Theorem 4, we consider two different scenarios. Recall that $B_{y,z}^{\alpha }=\alpha \delta _y+(1-\alpha )\delta _z$ with $y<z$ and $\alpha \in [0,1]$. The proof of the case that $T(B_{y_0,z_0}^{\alpha _0})(x_0)\in (0,1)$ for some $y_0<z_0$, $x_0\in \mathbb {R}$ and $\alpha _0\in (0,1)$ follows the similar argument as that of Theorem 4. We next consider the case that $T(B_{y,z}^{\alpha })(x)\in \{0,1\}$ for all $y<z$, $x\in \mathbb {R}$ and $\alpha \in (0,1)$. Following the same argument as in the proof of Theorem 4, we have that for some $c\in \mathbb {R}$, $T(F)=\delta _c$ for all $F\in {\mathcal {M}}$. We aim to extend this conclusion to $\mathcal {M}_0$. Note that commutation with $\mathcal {D}^R\cap \widehat{{\mathcal {D}}}$ implies that for any $d_1\in {\mathcal {F}}_D^{R}\cap \widehat{\mathcal {F}_D}$, there exist $d_2, d_3\in {\mathcal {F}}_D^R\cap \widehat{\mathcal {F}_D}$ such that $T_{d_2}\circ T=T\circ T_{d_1}$ and $T\circ T_{d_3}=T_{d_1}\circ T$. Let $d_1$ be a function in ${\mathcal {F}}_D^{R}\cap \widehat{\mathcal {F}_D}$ additionally satisfying $d_1(\varepsilon )=0$ and $d_1(1-\varepsilon )=1$ for some $\varepsilon \in (0,1/2)$. This implies $T_{d_1}(F)\in \mathcal {M}$ for all $F\in \mathcal {M}_0$. It follows from the conclusion on $\mathcal {M}$ that $T_{d_2}\circ T(F)=T\circ T_{d_1}(F)=\delta _c.$ Hence, we have for all $F\in \mathcal {M}_0$, $d_2(T(F)(c))=1$ and $d_2(T(F)(x))=0$ for all $x<c$. Moreover, by $T\circ T_{d_3}=T_{d_1}\circ T$, we have $T(d_3\circ F)(c)=d_1(T(F)(c))$. By freely choosing $d_1$, we have that if $T(F)(c)<1$, then there exists $d_3$ such that $T(d_3\circ F)(c)=d_1(T(F)(c))=0$. This implies $d_2(T(d_3\circ F)(c))=0$, which contradicts $d_2(T(F)(c))=1$ for all $F\in \mathcal {M}_0$. Hence $T(F)(c)=1$ for all $F\in \mathcal {M}_0$. If $T(F)(x)>0$ for some $x<c$, then by freely choosing $d_1$ we have that there exists some $d_3$ such that $T(d_3\circ F)(x)=d_1(T(F)(x))=1$. Then we have $d_2(T(d_3\circ F)(x))=1$, leading to a contradiction. Hence, $T(F)(x)=0$ for all $x<c$ and $F\in \mathcal {M}_0$. Combing the above conclusions, we obtain $T(F)=\delta _c$ for all $F\in \mathcal {M}_0$, which implies $T=T_d\circ T^c$, where $d\in {\mathcal {F}}_D^{R}\cap \widehat{\mathcal {F}_D}$ is strictly increasing and continuous. $\square$

## Footnotes

1 Obviously the choice may in general be multi-valued, but any selection would suffice.

2 It seems reasonable to assume that after-tax income is increasing, perhaps not strictly, with respect to pre-tax income.

3 This is not to say that the ultimate decision made by the decision maker is independent of the distributional transform—far from it. Rather, it reflects a model in which the economist understands exactly how probabilities are being transformed, independently of the underlying tax code.

4 That paper characterized a related class, but utilizing multiple axioms. Here, only one property is used to characterize probability distortions.

5 The two sets are related via a specific Galois connection, as we discuss in the conclusion. Galois connections are an order-theoretic notion of duality.

6 In the standard sense, where a function *f* is extended to sets by $f(A) = \{f(x):x\in A\}$.

7 An antitone Galois connection between two partially ordered sets $(A, \leqslant )$ and $(B, \leqslant )$ is a pair of two antitone functions $F: A \rightarrow B$ and $G: B \rightarrow A$, such that for all $a\in A$ and $b \in B$, we have $F(a) \leqslant b$ if and only if $a \leqslant G(b)$.
